# Impact of the COVID‐19 Pandemic on Influenza Circulation During the 2020/21 and 2021/22 Seasons, in Europe

**DOI:** 10.1111/irv.13297

**Published:** 2024-05-09

**Authors:** Mary A. Sinnathamby, Margaux M. I. Meslé, Piers Mook, The European Region Influenza Network Group Authorship, Richard Pebody, Silvia Bino, Iris Hasibra, Nune Bakunts, Romella Abovyan, Evgenia Khachtryan, Monika Redlberger‐Fritz, Nazifa Mursalova, Firuza Aliyeva, Veronika Vysotskaya, Natallia Shmialiova, Inna Karaban, Nathalie Bossuyt, Cyril Barbezange, Sanja Musa, Nina Rodić Vukmir, Amela Dedeić Ljubović, Dijana Baštinac, Nadezhda Vladimirov, Neli Korsun, Ivelina Trifonova, Irena Tabain, Goranka Petrović, Christos Karagiannis, Christos Haralambous, Helena Jirincova, Jan Kyncl, Ramona Trebbien, Lasse Skafte Vestergaard, Olga Sadikova, Irina Eero, Eliisa Metsoja, Niina Ikonen, Outi Lyytikäinen, Hanna Nohynek, Lucie Fournier, Caroline Guerrisi, Martine Valette, Ani Machablishvili, Silke Buda, Ralf Dürrwald, Georgia Gioula, Emmanouil Mary, Kassiani Mellou, Mónika Rózsa, Zsuzsanna Molnár, Brynja Armannsdottir, Guðrún Aspelund, Joan O’Donnell, Lisa Domegan, Jeff Connell, Michal Mandelboim, Aharona Glatman‐Freedman, Simona Puzelli, Anna Teresa Palamara, Francesco Maraglino, Smagulova Meiramgul Kanapiyanovna Smagulova, Aidar Sharipkhanuly Userbayev, Ariana Kalaveshi, Xhevat Jakupi, Zana Kaçaniku Gunga, D. S Otorbaeva, S. Zh Abdyldaeva, G. M Esengeldiev, Darja Vasiļevska, Kate Karolīna Tomašūna, Oksana Savicka, Greta Gargasiene, Svajune Muralyte, Trung‐Nguyen Nguyen, Joel Mossong, Tamir T. Abdelrahman, Tanya Melillo, Graziella Zahra, Jackie Melillo, Bozidarka Rakocevic, Zeljka Zekovic, Sanja Medenica, Marit de Lange, Adam Meijer, Dragan Kochinski, Golubinka Boshevska, Trinehessevik Paulsen, Karoline Bragstad, Lidia B. Brydak, Ewelina Hallmann, Karol Szymański, Ana Paula Rodrigues, Nuno Verdasca, Raquel Guiomar, Alina Druc, Mariana Apostol, Rodica Popescu, Odette Popovici, Mihaela Lazar, Andrey B. Komissarov, Artem Fadeev, Kirill Stolyarov, Jelena Protic, Mária Avdičová, Adriana Mečochová, Edita Staroňová, Maja Sočan, Katarina Prosenc, Nataša Berginc, Amparo Larrauri, Clara Mazagatos, Francisco Pozo, Emma Appelqvist, AnnaSara Carnahan, Ana Rita Goncalves Cabecinhas, Tania Spedaliero, Navaruz Jafarov, Tamanno Safarova, Barno Barotova, Tatyana Kovalchuk, Ayse Basak Altas, Emine Avci, Betul Ozdemir, Gurbangul Ovliyakulova, Tetiana Dykhanovska, Iryna Demchyshyna, Oksana Koshako, Conall Watson, Maria Zambon, Anissa Lakhani, Samantha Shepherd, Declan T. Bradley, Tanya Curran, Catherine Moore, Simon Cottrell, Lyudmila Kudasheva, Sultan Djemileva, Boris Pleshkov

**Affiliations:** ^1^ World Health Organization (WHO) Regional Office for Europe Copenhagen Denmark; ^2^ Institute of Public Health Tirana Albania; ^3^ National Center for Disease Control and Prevention Yerevan Armenia; ^4^ Medical University Vienna Austria; ^5^ Ministry of Health of Azerbaijan Baku Azerbaijan; ^6^ Republican Center for Hygiene, Epidemiology and Public Health Minsk Belarus; ^7^ Republican Research and Practical Center for Epidemiology and Microbiology Minsk Belarus; ^8^ Ministry of Public Health of the Republic of Belarus Minsk Belarus; ^9^ Epidemiology of Infectious Diseases Sciensano Brussels Belgium; ^10^ National Influenza Centre Sciensano Brussels Belgium; ^11^ Public Health Institute of the Federation of Bosnia and Herzegovina Sarajevo Bosnia and Herzegovina; ^12^ Public Health Institute of the Republic of Srpska Banja Luka Bosnia and Herzegovina; ^13^ Faculty of Medicine University of Banja Luka Banja Luka Bosnia and Herzegovina; ^14^ Clinical Center University of Sarajevo Sarajevo Bosnia and Herzegovina; ^15^ National Center of Infectious and Parasitic Diseases Sofia Bulgaria; ^16^ Croatian Institute of Public Health Zagreb Croatia; ^17^ Nicosia General Hospital Strovolos Cyprus; ^18^ Ministry of Health Nicosia Cyprus; ^19^ National Institute of Public Health Prague Czechia; ^20^ Statens Serum Institute Copenhagen Denmark; ^21^ The Estonian Health Board Tallinn Estonia; ^22^ Finnish Institute for Health and Welfare Helsinki Finland; ^23^ Sante publique France Paris France; ^24^ Centre National de Reference France Lyon France; ^25^ National Center for Disease Control and Public Health Georgia Tbilisi Georgia; ^26^ Robert Koch Institute Berlin Germany; ^27^ National Influenza Centre for N. Greece Aristotle University of Thessaloniki Thessaloniki Greece; ^28^ National Influenza Laboratory for S. Greece Hellenic Pasteur Institute Athens Greece; ^29^ National Public Health Organization Athens Greece; ^30^ National Public Health Center Budapest Hungary; ^31^ Landspitali University Hospital Reykjavik Iceland; ^32^ Centre for Health Security and Communicable Disease Control Reykjavik Iceland; ^33^ Health Service Executive, Health Protection Surveillance Centre Dublin Ireland; ^34^ National Virus Reference Laboratory University College Dublin Ireland; ^35^ Israel Ministry of Health Ramat Gan Israel; ^36^ Istituto Superiore di Sanità Rome Italy; ^37^ Ministry of Health (Italy) Rome Italy; ^38^ National Center for Public Health of the Ministry of Health of the Republic of Kazakhstan Almaty Republic of Kazakhstan; ^39^ National Institute of Public Health of Kosovo Pristina Kosovo; ^40^ Ministry of Health of the Kyrgyz Republic Bishkek Kyrgyzstan; ^41^ Center for Disease Prevention and Control of Latvia Riga Latvia; ^42^ National Public Health Center under the Ministry of Health Vilnius Lithuania; ^43^ National Public Health Surveillance Laboratory Vilnius Lithuania; ^44^ Laboratoire National De Santé Dudelange Luxembourg; ^45^ Ministry for Health Valletta Malta; ^46^ Institute of Public Health of Montenegro Podgorica Montenegro; ^47^ National Institute for Public Health and the Environment (RIVM) The Netherlands; ^48^ Institute of Public Health of the Republic of North Macedonia Skopje Republic of North Macedonia; ^49^ Norwegian Institute of Public Health Oslo Norway; ^50^ National Research Institute Warsaw Poland; ^51^ National Institute of Health Dr. Ricardo Jorge Lisbon Portugal; ^52^ National Agency for Public Health Chișinău Moldova; ^53^ National Institute of Public Health Romania Bucharest Romania; ^54^ Cantacuzino Military‐Medical Research and Development National Institute Bucharest Romania; ^55^ National Research Center for Epidemiology and Microbiology named after N. F. Gamalei of the Ministry of Health of Russia Moscow Russia; ^56^ Institute of Virology, Vaccines and Sera “Torlak” Belgrade Serbia; ^57^ Regional Public Health Authority Banská Bystrica Slovakia; ^58^ Public Health Authority of the Slovak Republic Bratslava Slovakia; ^59^ National Institute of Public Health Ljubljana Slovenia; ^60^ National Laboratory for Health, Environment and Food Maribor Slovenia; ^61^ National Center of Epidemiology, CIBERESP Carlos III Health Institute Madrid Spain; ^62^ National Center of Microbiology, CIBERESP Carlos III Health Institute Madrid Spain; ^63^ Public Health Agency of Sweden Solna Sweden; ^64^ University of Geneva Hospitals Geneva Switzerland; ^65^ Ministry of Health of the Republic of Tajikistan Dushanbe Tajikistan; ^66^ General Directorate of Public Health Türkiye Ministry of Health Ankara Türkiye; ^67^ Ministry of Health and Medical Industry of Turkmenistan Ashgabat Turkmenistan; ^68^ Public Health Center of the Ministry of Health of Ukraine Kyiv Ukraine; ^69^ UK Health Security Agency London England UK; ^70^ NHS Greater Glasgow and Clyde Glasgow Scotland UK; ^71^ Public Health Agency Northern Ireland Belfast Northern Ireland UK; ^72^ Belfast Health and Social Care Trust Belfast Northern Ireland UK; ^73^ Public Health Wales Cardiff Wales UK; ^74^ Sanitary and Epidemiological Welfare and Public Health Service of the Republic of Uzbekistan Tashkent Uzbekistan

**Keywords:** COVID‐19 pandemic, epidemiology, Europe, influenza, non‐sentinel, sentinel, severity, surveillance

## Abstract

**Background:**

The emergence of SARS‐CoV‐2 in late 2019 saw the implementation of public health and social measures (PHSM) by countries across Europe to reduce its transmission and impact on populations. Consequently, countries reported changes in influenza circulation and extensive disruptions to routine surveillance systems.

**Methods:**

We describe the epidemiology of influenza in Europe between Weeks 40/2020 and 39/2022 compared to the 2016/17 to 2019/20 seasons, to assess the impact of the COVID‐19 pandemic and PHSM on surveillance systems and influenza circulation.

**Results:**

Low detections of influenza were observed through primary care sentinel sources during seasonal influenza periods (Week 40 to 20); 56 (of 39,457 specimens tested; < 1% positivity) in 2020/21 and 7261 (of 64,153 specimens tested; 11% positivity) detections in 2021/22 were observed, compared to an average of 18,383 (of 50,544 specimens tested; 36% positivity) detections in 2016/17 to 2019/20. Similarly, 11 (of 19,989 specimens tested; < 1% positivity) and 1488 (of 23,636 specimens tested; 6% positivity) detections were reported through SARI surveillance sources in 2020/21 and 2021/22, respectively, compared to an average of 2850 (of 10,389 specimens tested; 27% positivity) detections in 2016/17 to 2019/20. However, the 2021/22 interseasonal period saw unusual increases in influenza detections across surveillance site types when PHSM were easing.

**Conclusion:**

In conclusion, findings suggest that the restriction and easing of PHSM measures were associated with variations in influenza detections. Our observations of out‐of‐season influenza activity highlight the importance of an integrated respiratory surveillance strategy to monitor circulating respiratory viruses throughout the year to inform optimal prevention and control strategies.

## 1 Introduction

Influenza surveillance is recognized to be of critical public health importance to monitor and assess the impact of seasonally circulating influenza viruses, which significantly contribute to global morbidity and mortality [1]. Following the emergence of the Severe Acute Respiratory Syndrome Corona Virus 2 (SARS‐CoV‐2) in 2020 (officially declared as a public health emergency of international concern on 30 January 2020) [2], a substantial decline in the circulation of a range of respiratory viruses, including influenza virus, was observed. This was notable through long‐established sentinel and non‐sentinel surveillance systems in countries, territories, and areas (hereafter referred to as countries) in the World Health Organization (WHO) European Region, the European Union (EU), and European Economic Area (EU/EEA) (hereafter referred to as Europe), in the 2019/20 and 2020/21 seasons [3, 4].

Influenza surveillance in the European Region is jointly coordinated by the European Centre for Disease Prevention and Control (ECDC) and the WHO Regional Office for Europe, where weekly epidemiological and virological influenza data are submitted by countries to The European Surveillance System database (TESSy; managed by ECDC). Regional surveillance data are used to determine the start, end, magnitude, and severity of the season as well as the dominant circulating influenza virus types, A subtypes and B lineages.

Sentinel surveillance systems remain the gold standard for the detection and monitoring of circulating respiratory viruses including influenza virus. In the European Region, sentinel surveillance for influenza is conducted by countries using a representative subset of primary care outpatient and, separately, hospital sites. These systems have centralized coordination and application of predefined case definitions such as influenza‐like illness (ILI) and/or acute respiratory infection (ARI) and severe acute respiratory infections (SARI), as described previously [5].

Existing sentinel systems in primary and secondary care have been negatively impacted by the COVID‐19 pandemic as a result of a spectrum of factors including limited access to health care, redistribution of patients and specimens to COVID‐19 testing centers, suspension of physical consultation in primary care, or limited capacity to maintain or enhance these systems given other pandemic‐related priorities [6].

To assess the impact of the COVID‐19 pandemic on surveillance systems, including laboratory confirmed hospitalizations for influenza, and influenza circulation, this study provides a descriptive epidemiological summary of influenza virus testing and detections in the European Region over the two main influenza seasons during the pandemic between Weeks 40/2020–2021 and 40/2021–20/2022 and the 2021 and 2022 interseasonal periods (Weeks 21–39) in comparison to the same in the previous four seasons (2016/17 to 2019/20).

## 2 Methods

This retrospective epidemiological analysis used data submitted to The European Surveillance System (TESSy) by countries in Europe. The influenza season is defined for the northern hemisphere as Week 40 in a given year to Week 20 of the following year. For the purposes of this analysis, each interseasonal period (ranging between Weeks 21 and 39 of a given year) was also included; however, it is of note that not all countries report or monitor influenza activity outside of the influenza seasonal weeks.

The study period ranges from Week 40/2020 to Week 39/2022 with comparisons to the four previous seasons (2016/17 to 2019/20), where appropriate.

### 2.1 Data Sources

Qualitative indicator intensity is a measure of influenza activity that considers the level of ILI and/or ARI rates as well as influenza virus detections and is reported based on an individual country assessment according to set definitions such as calculating and using their respective Moving Epidemic Method (MEM) thresholds per surveillance system [7].

Weekly aggregated data on number of sentinel tests and detections in primary care and hospital settings were extracted from TESSy during Week 40/2022 (7 October 2022), from reporting countries in Europe. Although, there is a total of 54 countries in Europe; the number of countries included for each surveillance system described in this study differs based on an individual country's ability to report data to each surveillance system; for example, one country may only have reported data for sentinel surveillance and not SARI surveillance; therefore; the number of countries reporting to SARI surveillance will differ.

The distribution of influenza virological data derived from specimens taken in sentinel primary care outpatient (from ILI or ARI cases) and hospital inpatients (from severe acute respiratory infections [SARI] cases) sites, separately, was summarized by week and where available by influenza virus type, A‐subtype or B‐lineage at regional level. A subset of countries additionally monitors laboratory‐confirmed influenza cases hospitalized in intensive care units (ICU) and/or other wards. Data from non‐sentinel sources (such as hospitals, schools, primary care facilities not involved in sentinel surveillance, or nursing homes and other institutions) were also summarized.

### 2.2 Statistical Methods

Circulating viruses were classified as dominant by surveillance systems if at least 10 specimens were tested and ≥ 60% of influenza viruses were identified as a given type (A or B), A‐subtype (A(H1) pdm09 [seasonal influenza subtype after the 2009 pandemic], A(H3)), or B‐lineage (B/Victoria, B/Yamagata) at regional level. If between 41% and 59% of viruses, inclusive, were assigned to more than one type or A‐subtype or B‐lineage, these viruses were classified as codominant. This methodology has been previously used and outlined in the TESSy guidance [5].

#### 2.2.1 Positivity (Proportion Positive)

Positivity was calculated as the number of weekly influenza virus‐positive specimens divided by the number of specimens tested for influenza virus, when at least 10 specimens were tested for a given week at regional level.

Epidemic influenza circulation is considered to have started when the first of two consecutive weeks with at least 10% of specimens from sentinel sources tested positive for influenza and the end of the epidemic as the last week with a percent positive of at least 10%.

#### 2.2.2 Stringency of Public Health and Social Measures (PHSM)

Stringency of PHSM was derived from the PHSM Severity Index [8], which was developed to capture a severity index for each country based on six standardized PHSM indicators (wearing of masks [face coverings], school closures, workplace closures, restrictions on gatherings, stay‐at‐home mandates, and international travel limitations) as reported by countries, to mitigate the transmission of COVID‐19. Stringency of PHSM was calculated [8] as the weekly mean percentage of the six composing measures from 15 January 2020 to 30 September 2022, with 0% defined as no restrictions imposed and 100% defined as all considered restrictions imposed. Data for the United Kingdom could not be disaggregated (England, Northern Ireland, Scotland, and Wales) for this analysis and was therefore considered as one member state of the WHO European Region.

## 3 Results

### 3.1 Seasonal Period (Week 40/2020 to 20/2021 and Week 40/2021 to 20/2022)

#### 3.1.1 Intensity Indicators

**FIGURE 1:**
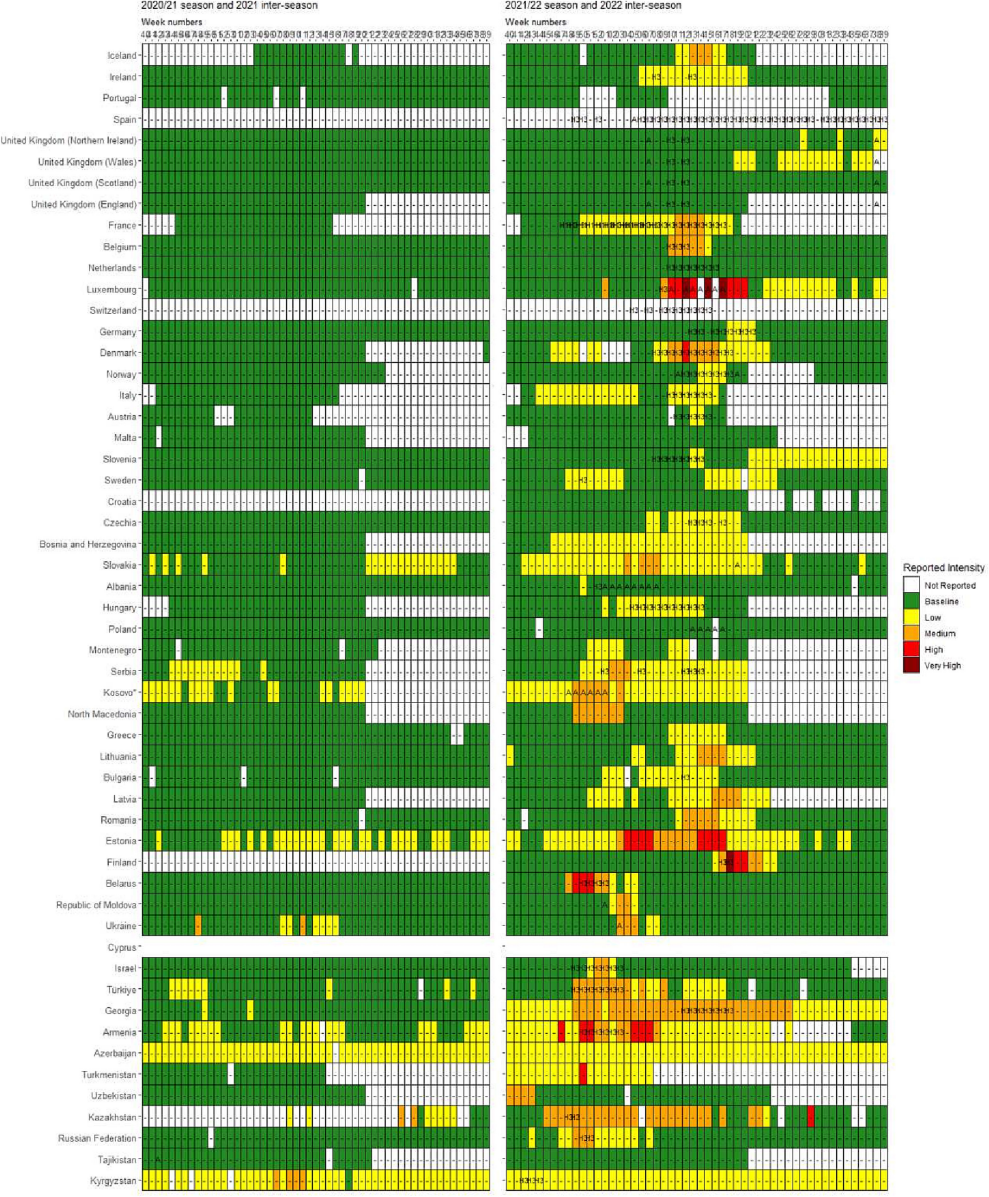
Qualitative indicator influenza intensity, by week and West (W) to East (E), between weeks 40/2020 and 39/2022, Europe. *Note:* Seasonal weeks: 40/2020 to 20/2021 and 40/2021 to 20/2022; interseasonal weeks: 21 to 39/2021 and 21 to 39/2022.

During the 2020/21 season, only 11 out of 54 countries (mainly in the eastern parts of the Region) reported at least 1 week of influenza intensity to be above baseline level, of which three countries (Kazakhstan, Kyrgyzstan, and Ukraine) reported at least 1 week of medium intensity in this time period (Figure 1). In comparison, during the 2021/22 season, 42 out of 54 countries reported at least 1 week of influenza intensity above baseline, including seven countries reporting at least 1 week of high intensity in Eastern Europe and two countries (Finland and Luxembourg) reporting very high intensity (Figure 1).

#### 3.1.2 Primary Care Sentinel Surveillance

**FIGURE 2:**
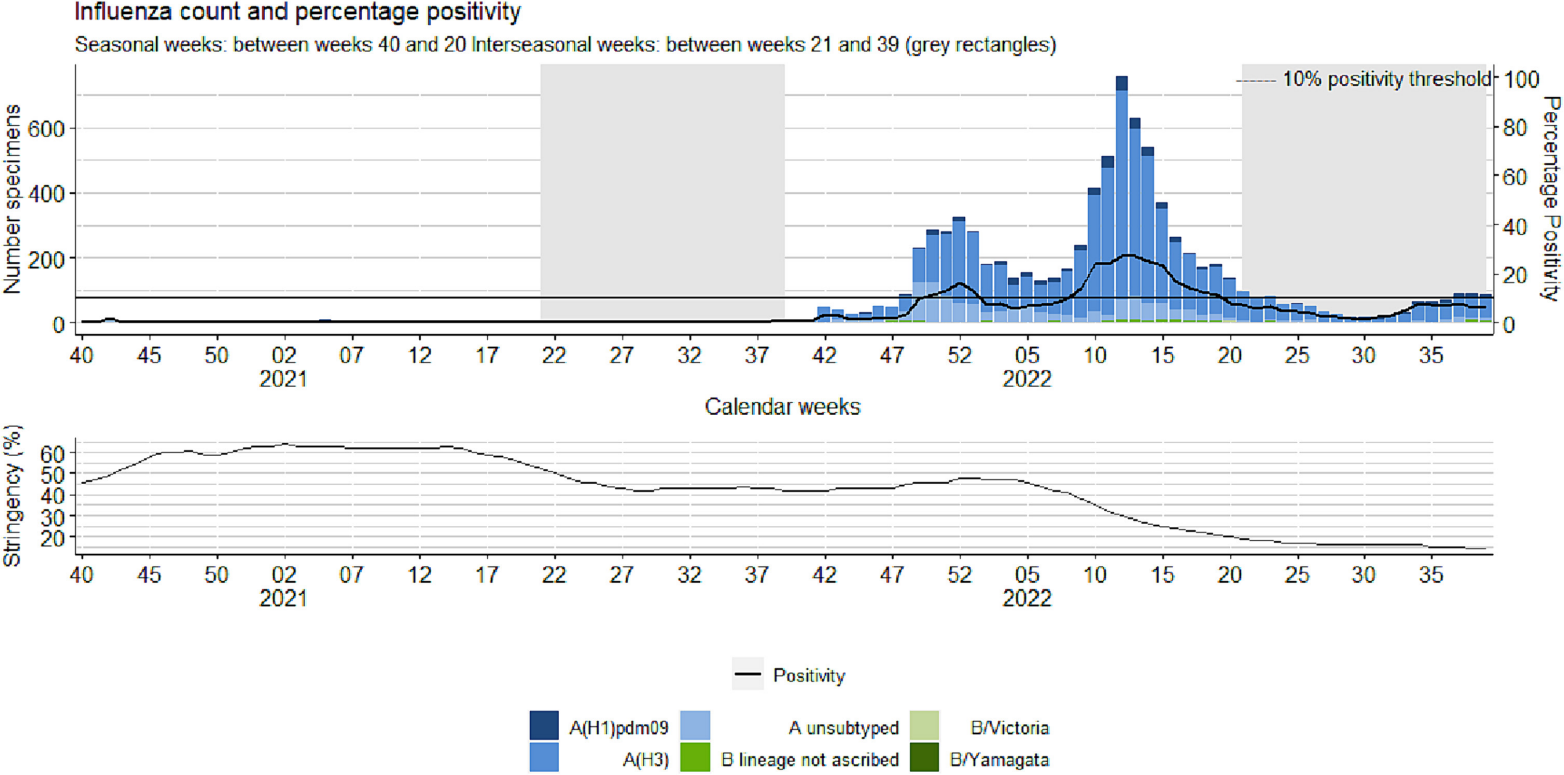
Weekly number of laboratory‐confirmed positive tested influenza specimens in sentinel primary care with percentage positivity (upper figure) and percentage of stringency of public health and social measures (PHSM) (lower graph), by week, between weeks 40/2020 and 39/2022, Europe.

Between Weeks 40/2020 and 20/2021, there was no notable seasonal trend in the circulation of influenza viruses compared to previous seasons with a small number (< 10 except in Week 42/2020) of sporadic weekly detections observed of both influenza virus types A and B through primary care sentinel surveillance sites. There were 40 influenza A virus (13 A(H1) pdm09, seven A(H3), 20 A [not subtyped]) and 16 influenza B virus (three B/Victoria lineage and 13 B with no known lineage; no B/Yamagata lineage) detections reported in these weeks from 44 countries (Tables 1 and 2 and Figures S1 and S3). The highest number of detections (*n* = 11, all influenza A [not subtyped]) was noted in Week 42/2020, and all reported by one country (Tajikistan) (Figures 2, S1 and S3). This is in stark contrast with the prior four seasons that had a mean of 18,382 detections and a range of 16,445 to 22,321 (Table 1).

**TABLE 1:** Number of countries reporting by reporting systems with total number of tested specimens and overall positivity for seasons 2020/21 and 2021/22 compared to the median and range of seasons 2016/17–2019/20, by season (weeks 40–20) and interseason (weeks 21–39), Europe.

	2021/22 season		2020/21 season		2016/17–2019/20 seasons			
	Seasonal period	Interseasonal period	Seasonal period	Interseasonal period	Season (mean)	Season (range)	Interseason (mean)	Interseason (range)
Sentinel surveillance								
Number of countries^a^	49	37	44	26	48	46–50	20.8	14–25
Specimens tested	64,153	20,709	39,457	7993	50,543.5	46,234–55,171	2110.5	1519–2904
Positive detections	7261 (11.3%)	1045 (5%)	56 (0.1%)	10 (0.1%)	18,382.5 (36.4%)	16,445–22,321	14.8 (0.7%)	3–37
Nonsentinel surveillance								
Number of countries^a^	46	33	41	31	47.2	45–48	23	16–26
Specimens tested	2,600,987	575,538	869,347	332,582	767,305.5	597,413–860,610	62,868.8	52,138–73,658
Positive detections	134,493	6650	867	360	176,715 (23%)	132,384–229,033	837 (1.3%)	48–1422
SARI surveillance								
Number of countries^a^	22	16	19	12	17	15–18	6	4–7
Specimens tested	23,636	7725	19,989	4146	10,389.0	9556–11,308	1122.8	474–1608
Positive detections	1488 (6.3%)	100 (1.3%)	11 (< 1%)	18 (0.4%)	2849.8 (27.4%)	2043–3645	7.8 (< 1%)	1–11

^a^ Reporting at least one specimen tested per season.

**TABLE 2:** Distribution of influenza virus (sub)types and lineages from primary care sentinel surveillance for 2020/21 and 2021/22 seasons and previous seasons for respective seasonal and interseasonal periods, Europe.

Subtype/lineage	2021/22 season		2020/21 season		2016/17–2019/20 seasons			
	Seasonal period	Interseasonal period	Seasonal period	Interseasonal period	Prev interseason (mean)	Seasonal period (mean)	Prev interseason (range)	Seasonal period (range)
Positive samples	7261 (11.3%)	1045 (5%)	56 (0.1%)	10 (0.1%)	14.8 (0.7%)	18,382.5 (36.4%)	3–37	16,445–22,321
Influenza A	7157 (99%)	1012 (97%)	40 (71%)	10 (100%)	8.0 (54%)	12,737.5 (69%)	1–14	8200–16,752
A(H1)	394 (7%)	85 (9%)	13 (65%)	0 (0%)	2.8 (40%)	4884.8 (42%)	0–7	149–8298
A(H3)	5626 (93%)	855 (91%)	7 (35%)	8 (100%)	4.2 (60%)	6864.5 (58%)	1–8	2650–13,375
Influenza A not subtyped	1137	72	20	2	1.0	988.2	0–2	579–1265
Influenza B	104 (1%)	33 (3%)	16 (29%)	0 (0%)	6.8 (46%)	5645.0 (31%)	1–23	248–14,121
B/Victoria	18	6	3	0	1.5	762.5	0–5	13–2492
B/Yamagata	0	0	0	0	0.0	1839.5	0–0	7–6943
B lineage unknown	86	27	13	0	5.3	3043.0	1–18	228–6978
Total samples tested	64,153	20,709	39,457	7993	2110.5	50,543.5	1519–2904	46,234–55,171

*Note:* For type percentage calculations, the denominator is total detections; for subtype and lineage, it is total influenza A subtyped and total influenza B lineage determined, respectively.

The highest positivity was also noted in Week 42/2020 at 2%, which means that a seasonal influenza epidemic was not declared as the 10% positivity threshold was not exceeded during any week of the seasonal period. This percentage positivity differs greatly from that observed over the previous four seasons (2016/17 to 2019/20) where the average peak positivity was calculated at 53% (range: 50%–59%) and often later in the seasonal period between Weeks 51 and 5 (Figure 5B).

The 2021/22 season was characterized by two peaks in influenza activity with positivity peaking at 16% in Week 52/2021 and 27% in Week 12/2022 (Figure 5B and Table 1). A total of 7261 influenza detections was noted with 99% characterized as influenza A, with the majority (5626) detected as A(H3), and 1% characterized as influenza B during this period in the Europe (Table 2). The first wave of activity was mainly noted in the Eastern parts of the Region, whereas activity in the Western parts of the region was mainly noted during the second wave in the latter weeks of the season, with Finland observing the greatest positivity at 80% during Week 17/2022 (Figure S1).

The largest number of specimens tested was recorded during the 2021/22 season, with a total of 64,153 specimens tested, which was a 21% increase in comparison to the average of 50,543.5 specimens tested over the previous seasons, 2016/17 to 2019/20 (Table 1). However, the range of weekly tests performed was more uniform across the season rather than being characterized by a peak in testing when positivity increased (Figure 5A). In contrast, the 2020/21 season saw a total of 39,457 specimens tested, a 28% decrease in comparison to the average of 50,543.5 specimens tested over the previous seasons, 2016/17 to 2019/20 (Table 1).

#### 3.1.3 Non‐sentinel Data

**FIGURE 3:**
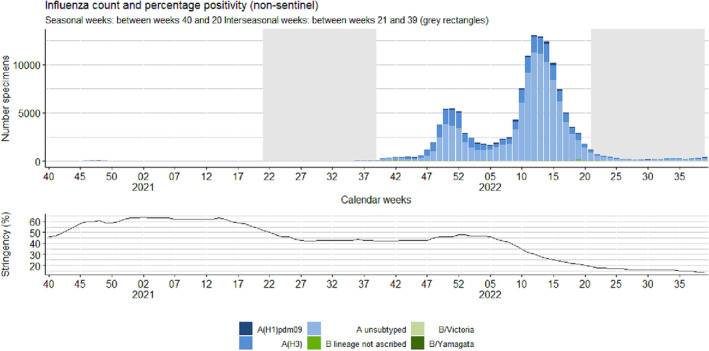
Weekly number of laboratory‐confirmed positive tested influenza specimens in non‐sentinel primary care with percentage positivity (upper figure) and percentage of stringency of public health and social measures (PHSM) (lower graph), by week, between weeks 40/2020 and 39/2022, Europe.

Between Weeks 40/2020 and 20/2021, there was no notable seasonal trend in the circulation of influenza viruses compared to previous seasons with a small number (≤ 50 except in Week 49/2020) of sporadic weekly detections observed of both influenza types A and B through primary care non‐sentinel surveillance sites. There were 436 influenza A (49 A(H3), 26 A(H1) pdm09, 361 A [not subtyped]) and 431 influenza B (12 B/Victoria lineage, one B/Yamagata lineage [derived from LAIV vaccines] and 418 B with no known lineage) detections reported in these weeks from 41 countries (Tables 1 and 3). A total of 24 countries reported at least one detection per week for the season; the highest number of detections (*n* = 50, 27 type B and 23 type A) was noted in Week 49/2020, of which 72% (*n* = 36) was from United Kingdom (England) (Figure 3). This is in stark contrast with pre–COVID‐19 pandemic seasons where the number of detections ranged between 132,384 and 229,033.

**TABLE 3:** Distribution of influenza virus (sub)types and lineages from non‐sentinel surveillance for 2020/21 and 2021/22 seasons and previous seasons for respective seasonal and interseasonal periods, Europe.

Subtype/lineage	2021/22 season		2020/21 season		2016/17–2019/20 seasons			
	Seasonal period	Interseasonal period	Seasonal period	Interseasonal period	Prev interseason (mean)	Seasonal period (mean)	Prev interseason (range)	Seasonal period (range)
Positive samples	134,493	6650	867	360	837	176,715	48–1422	132,384–229,033
Influenza A	132,117 (98%)	6267 (94%)	436 (50%)	301 (84%)	503.2 (60%)	130,557.5 (74%)	30–1147	102,528–194,096
A(H1)	2631 (9%)	398 (18%)	26 (35%)	6 (3%)	76.2 (25%)	18,588.2 (42%)	4–138	420–36,515
A(H3)	28,036 (91%)	1869 (82%)	49 (65%)	222 (97%)	226.8 (75%)	25,729.8 (58%)	6–567	16,638–40,086
Influenza A not subtyped	101,450	4000	361	73	200.2	86,239.5	20–445	65,107–131,578
Influenza B	2376 (2%)	383 (6%)	431 (50%)	59 (16%)	333.8 (40%)	46,157.5 (26%)	18–1032	2082–126,505
B/Victoria	98	32	12	1	9.5	675.8	0–23	46–2067
B/Yamagata	2	0	1	0	47.2	2644.8	0–179	65–8919
B lineage unknown	2276	351	418	58	277.1	42,836.9	18–840	1958–117,388
Total samples tested	2,600,987	575,538	869,347	332,582	62,868.8	767,305.5	52,138–73,658	597,413–860,610

*Note:* For subtype and lineage percentage calculations, the denominator is the total influenza A subtyped and total influenza B lineage determined, respectively; as not all countries have a true non‐sentinel testing denominator, no percentage calculations for total tested are shown.

The 2021/22 season was also characterized by two peaks in influenza non‐sentinel activity with detections peaking in Week 51/2021 (*n* = 5420) and in Week 12/2022 (*n* = 12,988), during each respective peak, albeit not to the same level as the average noted across the previous four seasons (range of 132,384 to 229,033) (Figure 5C and Table 1). Influenza A viruses were dominant in this season, accounting for 98% of detections, of which 91% was characterized as A(H3) (Table 3). The countries most affected by each wave were not as distinct as seen in sentinel surveillance, with some countries like Finland, Montenegro, Republic of Moldova, Spain, Sweden, and Tajikistan experiencing two distinct waves of activity (Figure S1).

The first peak of detections (Week 51/2021) saw 15 countries reporting at least 10 detections, with Sweden and Russian Federation reporting the most, with 1851 and 1320, respectively. The second peak of detections (Week 12/2022) saw 29 countries reporting detections, with Denmark and France reported 3214 and 2016 detections, respectively.

The countries with the largest number of influenza B virus detections were Netherlands (Kingdom of the) (*n* = 202), Russian Federation (*n* = 256), and the United Kingdom (England) (*n* = 950), with the Russian Federation reporting most of its detections (*n* = 23, 79% of detections) as type B (no lineage ascribed). However, the biggest proportion of type B detections was reported by Kazakhstan (*n* = 23, 64% of detections), followed by the United Kingdom (Northern Ireland) (*n* = 125, 22% of detections) and Poland (*n* = 33, 13% of detections) during the 2021/22 season.

#### 3.1.4 SARI Surveillance

**FIGURE 4:**
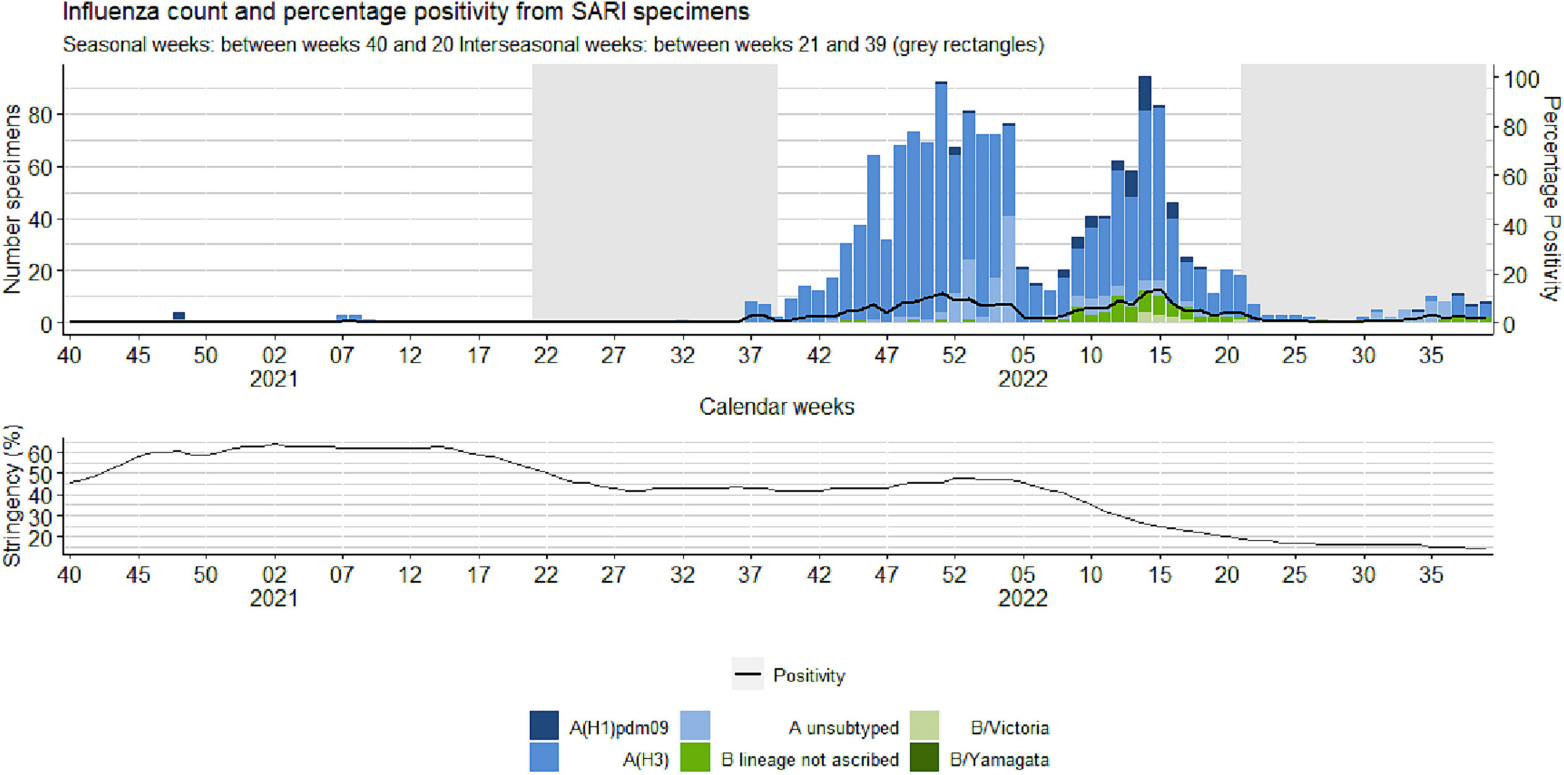
Weekly number of laboratory‐confirmed positive tested influenza specimens in hospitalized patients with severe acute respiratory infections (SARI) and percentage positivity (upper graph) and percentage of stringency of public health and social measures (PHSM) (lower graph), by week, between weeks 40/2020 and 39/2022, Europe.

In line with observations from the primary care surveillance sites, between Weeks 40/2020 and 20/2021, very low influenza virus detections were noted through SARI surveillance sites reported by 19 countries. A total of 11 (< 1% positivity) influenza A virus detections was reported from two countries (Armenia and Ukraine) (seven A(H3), three A(H1) pdm09, and one A [not subtyped]) and no influenza B virus detections reported (Table 4 and Figure S2). Week 48/2020 was noted to be the week with the most detections during the 2020/21 season where four influenza A virus detections (three A(H1) pdm09 and one A [not subtyped]) were reported by one country (Ukraine) (Figures 4 and S2).

**TABLE 4:** Influenza viral virus distribution by (sub)types and lineage from severe acute respiratory infections (SARI) surveillance for 2020/21 and 2021/22 seasons and previous seasons for respective seasonal and interseasonal periods, Europe.

Subtype/lineage	2021/22 season		2020/21 season		2016/17–2019/20 seasons			
	Seasonal period	Interseasonal period	Seasonal period	Interseasonal period	Prev interseason (mean)	Seasonal period (mean)	Prev interseason (range)	Seasonal period (range)
Positive samples	1488 (6.3%)	100 (1.3%)	11 (0.1%)	18 (0.4%)	7.8 (0.7%)	2849.8 (27.4%)	1–11	2043–3645
Influenza A	1411 (95%)	86 (86%)	11 (100%)	18 (100%)	4.0 (51%)	1996.2 (70%)	0–10	893–2770
A(H1)	59 (5%)	4 (7%)	3 (30%)	0 (0%)	2.0 (50%)	842.2 (46%)	0–7	7–1978
A(H3)	1208 (95%)	52 (93%)	7 (70%)	17 (100%)	2.0 (50%)	975.8 (54%)	0–3	301–2564
Influenza A not subtyped	144	30	1	1	0.0	178.2	0–0	44–267
Influenza B	77 (5%)	14 (14%)	0 (0%)	0 (0%)	3.8 (49%)	853.5 (30%)	0–8	32–1304
B/Victoria	10	2	0	0	0.2	214.8	0–1	0–673
B/Yamagata	0	0	0	0	0	123	0–0	1–341
B lineage unknown	67	12	0	0	3.6	515.7	0–8	31–770
Total samples tested	23,636	7725	19,989	4146	1122.8	10,389.0	474–1608	9556–11,308

*Note:* For type percentage calculations, the denominator is total detections; for subtype and lineage, it is total influenza A subtyped and total influenza B lineage determined, respectively.

**FIGURE 5:**
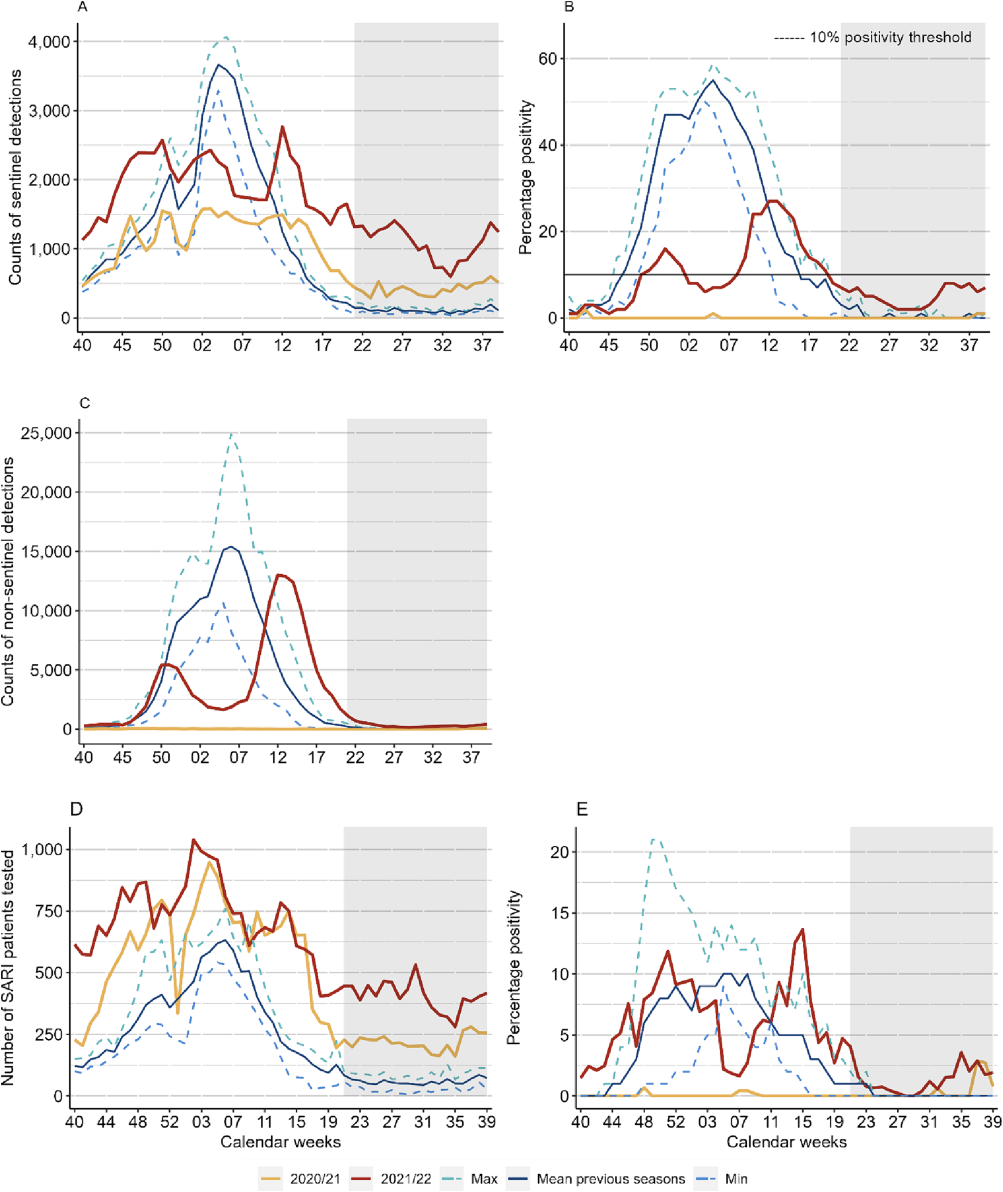
Top row: count of specimens detected (A) and percentage positivity (B) of influenza specimens tested per week from sentinel sources compared to the mean, minimum (Min), and maximum (Max) from pre–COVID‐19 pandemic seasons; middle row: count of non‐sentinel influenza detections compared to the mean, minimum (Min), and maximum (Max) from pre–COVID‐19 pandemic seasons (C); bottom row: count of specimens detected for influenza (D) and percentage positivity (E) of patients tested per week from SARI sites compared to the mean, minimum (Min), and maximum (Max) from pre–COVID‐19 pandemic seasons, Europe. *Note:* Percentage positivity was calculated when at least 10 specimens were tested.

In contrast, a total of 1488 (6% positivity) detections was reported from 22 countries during the 2021/22 season, with influenza A viruses (95%) accounting for most detections of which 96% were influenza A(H3) (Tables 2 and 4 and Figure 4). Additionally, of the 77 influenza B virus detections, 10 were attributed to the B/Victoria lineage and none to the B/Yamagata lineage (Table 4). These detections were from 22 countries, of which both Lithuania and Serbia recorded the highest overall percentage positivity of 36% (Figure S4). Armenia recorded a peak of 70% (50/71) positivity (Week 51/2021), much higher than in any previous season (Figure S4). Similarly, to sentinel surveillance, SARI detections saw two waves of activity, with a first peak of positivity in Week 51/2021 (92 detections; 12% positivity) and in Week 15/2022 (83 detections; 14% positivity) (Figures 4 and 5E).

During the 2021/22 season, the number of patients tested was higher than the 2020/21 season, with a total of 23,636 tests performed (Table 1). A peak in testing was observed in Week 2/2022, with 1039 tests compared to an average of 463.8 in prior seasons for the same week (Figure 5D).

The weekly number of SARI patients tested for influenza in both seasons was continuously greater than that of the average across the previous four seasons. During the 2020/21 season, a peak of 947 patients tested was noted in Week 4/2021 in comparison to the average peak of 583.8 tests (range: 501–610) in the same week in prior seasons mainly reported by Turkmenistan and Albania (Figure 5D).

In the 14 countries where a comparison was possible, Republic of Moldova and Ukraine were the only countries where SARI positivity peaked later compared to sentinel positivity. In the other 12 countries, peaks of positivity in both systems occurred at a similar time (Figures S2 and S4).

#### 3.1.5 Laboratory‐Confirmed Hospitalizations

During the 2020/21 season, three laboratory‐confirmed influenza hospitalizations (one influenza A(H1), one influenza A [not subtyped]. and one influenza B [no lineage ascribed]) from ICU wards were reported from three countries (Czechia, Sweden, and Ukraine) with no clear age group distinction (Table 5). Only two laboratory‐confirmed infections were reported from non‐ICU wards during the seasonal period (from Ukraine); both were patients infected with influenza A(H1) pdm09 viruses and aged between 15 and 64 years.

**TABLE 5:** Influenza viral virus distribution by (sub)types and lineage from severe acute respiratory infections (SARI) surveillance for 2020/21 and 2021/22 seasons and previous seasons for respective seasonal and interseasonal periods, Europe.

Hospital ward	Variable	2021/22 season	2022 interseason	2020/21 season	2021 interseason
ICU	A(H1)pdm09	59	8	1	0
	A(H3)	93	3	0	0
	A unsubtyped	580	60	1	0
	B	7	8	1	1
	Total (subtypes)	739	79	3	1
	00–04 years	58	1	0	0
	05–14 years	53	1	1	0
	15–64 years	250	2	1	1
	65+ years	198	5	1	0
	Age unknown	180	70	0	0
	Total (ages)	739	79	3	1
Other wards	A(H1)pdm09	3	3	2	0
	A(H3)	155	8	0	0
	A unsubtyped	413	72	0	0
	B	3	2	0	0
	Total (subtypes)	574	85	2	0
	00–04 years	72	15	0	0
	05–14 years	38	3	0	0
	15–64 years	223	36	2	0
	65+ years	241	31	0	0
	Total (ages)	574	85	2	0

In contrast, during the 2021/22 season, 739 laboratory‐confirmed hospitalizations from ICU wards (from Czechia, France, Ireland, Sweden, and the United Kingdom [England]) and 574 from non‐ICU wards (from Czechia, Ireland, and Ukraine) were reported. Of those reported from ICU wards, 732 (99%) were type A (of which 93 [13%] were A(H3), 59 [8%] were A(H1) pdm09, and 580 [79%] were not subtyped), and 7 were type B (no lineage ascribed). Of those with known age (559 patients, 69%), 250 (45%) were aged between 15 and 64 years, 198 (35%) were aged 65 years and older, 58 (10%) were aged 4 years or younger, and 53 (9%) were aged between 5 and 14 years (Table 5). Of the 574 patients reported from the non‐ICU wards, only three (< 1%) were reported to be infected with type B viruses (no lineage ascribed), and of the type A viruses, 155 (27%) were A(H3), three (1%) were A(H1) pdm09, and 413 (72%) were not subtyped. Of these non‐ICU patients, 241 (42%) were aged 65 years and older, 223 (39%) were aged between 15 and 64 years, 72 (13%) were aged four and younger, and 38 (7%) were aged between 5 and 14 years (Table 5). The largest number of cases from ICU wards (*n* = 81) were detected in Week 15/2022, but in Week 10/2022 from non‐ICU wards (*n* = 93).

### 3.2 Interseasonal Period (Weeks 21 to 39/2021 and Weeks 21 to 39/2022)

#### 3.2.1 Intensity Indicators

During the 2020/21 interseasonal period, only seven out of 31 countries reported influenza intensity to be above baseline level, of which Kazakhstan reported at least 1 week of medium intensity in this time period (Figure 1). In comparison, during the 2021/22 season, nine out of 34 countries reported influenza intensity above baseline, including Kazakhstan that reported very high intensity in 1 week (Figure 1).

#### 3.2.2 Primary Care Sentinel Surveillance

Between Weeks 21 and 39/2021, there were 10 influenza type A viruses (eight A(H3) and two A [not subtyped]) reported from three countries (France, Germany, and Kyrgyzstan) and no type B virus detections (Table 2 and Figures 3 and S3). This total number of type A virus detections was lower than the average number of detections seen during prior interseasonal periods, but the lack of detection of type B viruses contrasted with their detection in prior interseasonal periods (Table 2). The total number of primary care sentinel specimens tested (*n* = 7993) for influenza virus during this period was greater than those observed in the average number of specimens tested in the previous four seasons (average: 2110.5) (Tables 1 and 2). Week 39/2021 saw the highest number of detections during the 2021 interseason with 5 detections (all influenza A(H3)); all detections were reported from Kyrgyzstan. The overall positivity in Week 39/2021 was 22%, which was greater than the average positivity of < 1% noted in previous seasons for this period (Figure 5B).

Between Weeks 21 and 39/2022, a total of 1045 influenza virus detections was reported, with an overall positivity of 5%, which was higher than the average number of detections and positivity (<1%) from any prior interseasonal periods. The majority (97%) of these detections were type A viruses, of which A(H3) accounted for 91% (*n* = 855). Of the 33 type B viruses identified, six were B/Victoria and none of those subtyped were B/Yamagata (Tables 1 and 2). The largest number of detections was recorded in Week 21/2022 (Figure 2), with 96 detections (of which 91 were type A(H3), two were A(H1) pdm09, and two were not subtyped) reported from 12 countries and the majority (*n* = 62, 65%) was identified in Spain. From Week 34 to 39/2022, percentage positivity ranged between 6% and 8%, which was higher than the same weeks in any previous seasons (Figure 5B).

#### 3.2.3 Non‐sentinel Data

Between Weeks 21 and 39/2021, there were 301 (84% of detections) influenza type A viruses (222 A(H3), six A(H1) pdm09, and 73 A [not subtyped]) reported from 31 countries and 59 type B virus detections (only one was ascribed to a lineage, and it was B/Victoria) (Tables 1 and 3 and Figure 3). This total number of type A virus detections was within range of the average number of detections seen during prior interseasonal periods (Table 3). Week 37/2021 saw the highest number of detections during the 2021 interseason with 88 detections (52 influenza A(H3), one A(H1) pdm09, 20 influenza A unsub typed and 15 type B viruses [no lineage ascribed]) with the majority of detections (*n* = 43, all A(H3)) reported from Croatia. The maximum number of detections previously seen in pre–COVID‐19 pandemic seasons during Week 37 was 60 (Figure 5C).

Between Weeks 21 and 39/2022, a total of 6570 influenza virus detections was reported, which was higher than the average number of detections from any prior interseasonal periods (maximum number of detections = 1422). The majority (94%) of these detections was type A viruses, of which A(H3) accounted for 83% (*n* = 1846). Of the 368 type B viruses identified, 32 were B/Victoria, and none were B/Yamagata (Tables 1 and 3). The largest number of detections was recorded in Week 21/2022 (Figure 3), with 1145 detections (of which 288 were type A(H3), 14 were A(H1) pdm09, 784 were not subtyped, and 59 were type B [three were B/Victoria]) reported from 21 countries and the largest proportion (*n* = 339, 30%) were identified in Norway. Between Weeks 25 and 39/2022, the number of positive detections ranged between 152 and 365, which was higher than the same weeks in any of the four pre–COVID‐19 pandemic seasons (Figure 3).

#### 3.2.4 SARI Surveillance

Between Weeks 21 and 39/2021, there were no type B virus detections and 18 influenza virus type A detections (17 A(H3), and one A (not subtyped) (Tables 3 and 4) reported from three countries (Croatia, Kyrgyzstan, and Russian Federation). The average weekly number of SARI patients tested for influenza during this period was greater than the average number of patients tested in previous seasons: 218.2 specimens tested compared to an average of 59 per week in prior seasons. The largest number of patients (*n* = 280) were tested in Week 37/2021 in comparison to an average of 69.8 tests (range: 28–105) in Week 37 during the previous four seasons (Figure 5B).

Between Weeks 21 and 39/2022, a total of 100 detections was reported, of which the majority (86%) was type A (52 were A(H3), four were A(H1) pdm09, and 30 were not subtyped) and 14 were type B (two B/Victoria, 12 did not have a lineage ascribed) (Tables 2 and 4). These detections were reported from seven countries (Georgia, Ireland, Kazakhstan, Kyrgyzstan, Malta, Russian Federation, and Uzbekistan) (Figure S4). A peak of 4% percentage positivity was seen in Week 35/2022, with 10 detections from 280 tests. The average number of weekly patients tested for the 2022 interseason was 406.6, higher than in any prior season, with a peak in testing in Week 30/2022 (*n* = 532 patients tested performed) (Figure 5).

#### 3.2.5 Laboratory Confirmed Hospitalizations

Only one case was identified from ICU wards during the 2021 interseasonal period. The patient was infected with a type B virus (no lineage ascribed). No cases were reported from other wards during this period (Table 5).

During the 2022 interseasonal period, 79 cases were reported from ICU wards from four countries (Czechia, Ireland, Sweden, and United Kingdom [England]). The majority of which (*n* = 71, 90%) was infected with type A viruses (60 were not subtyped, eight were A(H1) pdm09, and three were A(H3)), and eight were infected with type B viruses (no lineage ascribed). Of the cases with known age groups (*n* = 9), five were aged 65 years and older, two were aged between 15 and 64 years, one was aged between 5 and 14 years, and one was younger than 4 years (Table 5). During the same period, 85 patients were identified from other wards, all reported from Ireland. Of these patients, 83 were infected with type A viruses (eight were infected with A(H3) and three with A(H1) pdm09), and two were infected with type B viruses (no lineage ascribed). Of these 85 patients, 67 were aged 15 years and older (36 were aged between 15 and 64 years and 31 were aged 65 years and older), three were aged 5 to 14 years, and 15 were 4 four or younger).

## 4 Discussion

Our study described substantially fewer detections and circulation of influenza during the 2020/21 (Week 40 to 20) and during the 2021/22 seasonal period despite an increase testing in this season, in comparison to those observed in the previous four influenza seasonal periods (2016/17 to 2019/20) in Europe. This further builds on evidence seen mid‐season of 2020/21 [3]. We also highlight that despite the subsequent resurgence in influenza activity in 2021/22 compared to the 2020/21 season, the circulation and timing were different to typical influenza annual epidemic activity observed before the COVID‐19 pandemic and seasons following the 2009 influenza pandemic. The study saw additional unusual interseasonal detections reported from sentinel, non‐sentinel, and SARI surveillance sites in 2022.

Our findings of low or no detections of influenza viruses through both primary and SARI systems in 2020/21 coincided with increased transmission of SARS‐CoV‐2 during the 2020/21 seasonal period and high levels of PHSM stringency implemented to reduce the transmission of SARS‐CoV‐2, but which also disrupted influenza virus transmission. Indeed, since the declaration of the COVID‐19 pandemic in March 2020 and subsequent implementation of PHSM across the globe, decreases in influenza virus detections have been noted in the latter part of the 2019/20 influenza season across Europe, a trend that was still evident mid‐season of 2020/21 [2, 3, 9, 10]. The findings of decreased detections of influenza during the 2020/21 season are consistent with those observed in other countries of the Northern as well as then the Southern hemisphere, with influenza positivity not exceeding 10% throughout each hemisphere's seasonal period [11, 12, 13, 14]. This is also evidenced through our findings of decreased influenza virus detections when the PHSM were at their most stringent denoting the impact of the SARS‐CoV‐2 waves on influenza detections in the Region. With the reduced stringency of PHSM during the 2021/22 season, atypical late influenza activity was detected with two waves of activity, although this circulation was still lower than during the 2016/17 to 2019/20 seasons. Further aberrant circulation of influenza can still be anticipated.

Additionally, our findings highlighted a mix of influenza subtypes circulating, and we noted that only one influenza B/Yamagata lineage virus derived from LAIV vaccines was detected throughout the study periods. Possible extinction of B/Yamagata has been highlighted before, and in line with this, since September 2023, WHO has recommended the exclusion of a B/Yamagata lineage antigen in seasonal influenza vaccines [15, 16]

It is also significant to note that our study of reduced influenza virus detections coincided with high numbers of SARS‐CoV‐2 detections during the seasonal period in the SARI data, and an increase in influenza virus detections was noted when the circulation of SARS‐CoV‐2 detections decreased in both interseasonal periods [17]. This was particularly evidenced through SARI sentinel sites during the interseasonal period. It has been suggested these observations may be due to viral interference [18]. Further work is required to understand this phenomenon better.

The reduction in testing activity could be one factor that contributed to a lower detection of influenza viruses. Most probably, a true reduction in influenza virus transmission occurred because some countries sustained their sentinel surveillance at the same level as before. We demonstrate ongoing testing for influenza through a range of surveillance systems, with actually greater number of specimens tested for influenza than several seasons before, and through both primary care sentinel and SARI surveillance schemes during the interseasonal period in 2022. This observation of increased testing may have been due to the increased use of multiplex assays to test for influenza viruses, SARS‐CoV‐2 and RSV. There was also the increased need to detect SARS‐CoV‐2 and its variants at a time when PHSM were relaxed. Nonetheless, it is important to note that there were changes in the number of reporting countries during the pandemic in comparison to pre‐pandemic seasons [19]. The main factor for the reduced transmission thus appears to be the impact of PHSM, across the Region. These measures aimed to mitigate the increasing spread of SARS‐CoV‐2 variants, Alpha (B.1.1.529) and Beta (B.1.351) detected in December 2020; however, as measures were relaxed in the 2021/22 season and at a time when the Omicron variant, with greater transmissibility, began circulating and as it was also usually a time of seasonal circulation of influenza viruses, the implementation of PHSM would have hypothetically also naturally reduced transmission of influenza virus. Potential viral interference and competition of both influenza and SARS‐CoV‐2 could have also been a factor [17]. Further work is required to disentangle these effects.

The reduced circulation of influenza virus for a prolonged period poses several uncertainties and implications for future seasons. First, the reduced/lack of exposure to influenza viruses increases susceptibility among populations, particularly those in younger age groups, within whom late first exposure to such viruses, may impact future immune response, but also in older age‐groups in whom immunity may then have waned. This could lead to a surge in rates of influenza once its circulation resumes and/or it co‐circulate with other respiratory viruses [20]. Indeed, the 2022/23 season has seen an unexpectedly early influenza season [21]. Second, the lack of circulation may have an impact on virus characterization for the annual recommendations of influenza vaccines' composition as predictions are heavily reliant on laboratory information from characterization of currently circulating viruses [6]. These characteristics, therefore, have been based on a reduced sample of circulating viruses available increasing the risk of suboptimal vaccine effectiveness. This issue is less of a concern now since the circulation of influenza has increased in the 2022/23 season and in subsequent seasons.

Some limitations to this study should be considered. First, it is important to highlight the influence of the COVID‐19 pandemic on the varying degrees of disruption to national sentinel surveillance systems, particularly influenza‐specific indicators such as ILI and ARI rates, due to changes in health seeking behaviors and limitations in the capacity of sites to receive cases and take specimens, impacting their ability to monitor respiratory viruses, including RSV. Second, not all countries who collate sentinel surveillance data report these data through the TESSy [19]. Furthermore, some countries do not maintain all‐year‐around surveillance and, therefore, may not have reported data for the interseasonal periods, introducing the likelihood of underestimating our findings. Third, not all countries have fully implemented an integrated approach with the inclusion of influenza and SARS‐CoV‐2 testing in NICs and, therefore, will still note varying testing numbers. Despite this, findings from sentinel surveillance, which has been considered to be the gold standard for the monitoring of influenza, correlate with those from non‐sentinel sources, as described earlier [3]. Lastly, there is potential for differential reporting across countries, due to variations in population coverage; for example, there may have been an increase in participating surveillance sites due to the COVID‐19 pandemic, sampling approaches, for example, the use of antigen testing impacting health seeking behaviors during the COVID‐19 pandemic and laboratory techniques used (for example, multiplex assays).

In conclusion, the 2020/21 season observed exceptionally low detections of influenza virus, despite elevated testing, this was followed by unusual activity during the 2021 interseasonal period when SARS‐CoV‐2 circulation was low. The easing of PHSM was associated with a rise in influenza virus detections during the 2021/22 season in many countries. The circulation and timing of influenza activity during the 2021/22 season are not comparable to any influenza annual epidemic activity observed before the COVID‐19 pandemic.

As countries move towards integrating surveillance of SARS‐CoV‐2, influenza, and other relevant respiratory viruses following the ECDC and WHO guidance from October 2020, underlying systems might change and reported data might not be comparable to historical data [3, 4, 22, 23, 24]. Further work is also needed to understand the recent lack of circulation of influenza B/Yamagata lineage, which has had implications on vaccine composition [15, 16]. Our study has highlighted the importance of resourcing, strengthening, and implementing integrated surveillance across the region for the most commonly circulating respiratory viruses throughout the year, to identify unusual out of season detections as we move from the acute phase of the COVID‐19 pandemic. It is, therefore, vital for countries to continue to plan towards the implementation of robust and agile integrated respiratory disease surveillance in line with the WHO European Region and ECDC guidelines [23] to vigilantly and simultaneously survey, sequence, and report the circulation of the most commonly circulating respiratory pathogens such as influenza, RSV, and SARS‐CoV‐2 [8].

## Author Contributions

**Mary A. Sinnathamby:** conceptualization, formal analysis, methodology, supervision, writing–original draft, writing–review and editing. **Margaux M. I. Meslé:** data curation, formal analysis, methodology, writing–original draft, writing–review and editing. **Piers Mook:** conceptualization, formal analysis, methodology, supervision, writing–original draft, writing–review and editing. **Richard Pebody:** conceptualization, supervision, writing–review and editing.

## Conflicts of Interest

The authors declare no conflicts of interest.

## Supporting information


**Figure S1.** Individual country‐level weekly number and percentage positivity reported for influenza detections through sentinel primary care surveillance, between Week 40/2020 and Week 39/2021, Europe.
**Figure S2.** Individual country‐level weekly number and percentage positivity reported for influenza detections through SARI surveillance, between Week 40/2020 and Week 39/2021, Europe.
**Figure S3.** Individual country‐level percentage positivity for influenza, Week 40/2020 and 39/2021 in comparison with the mean, minimum, maximum number of specimens in the previous four seasons (Week 40 to 39, 2015/16 to 2019/20) through sentinel primary care surveillance, Europe.
**Figure S4.** Individual country‐level percentage positivity for influenza, Week 40/2020 and 39/2021 in comparison with the mean, minimum, maximum number of specimens in the previous four seasons (Week 40 to 39, 2015/16 to 2019/20) through SARI surveillance, Europe.

## Data Availability

TESSy data are available upon request (https://www.ecdc.europa.eu/en/publications‐data/european‐surveillance‐system‐tessy).
