# Supplementary material for: Impact of the COVID‐19 Pandemic on Influenza Circulation During the 2020/21 and 2021/22 Seasons, in Europe
Source: Influenza Other Respir Viruses. 2024 May 9;18(5):e13297. doi: 10.1111/irv.13297 (PMC11081882; doi:10.1111/irv.13297)

**Manuscript: Impact of the COVID-19 pandemic on influenza circulation during the 2020/21 and 2021/22 seasons, in Europe**

**Supplementary Figures**

Supplementary Figure 1 (SF1). Individual country-level weekly number and percentage positivity reported for influenza detections through sentinel primary care surveillance, between week 40/2020 and week 39/2021, Europe

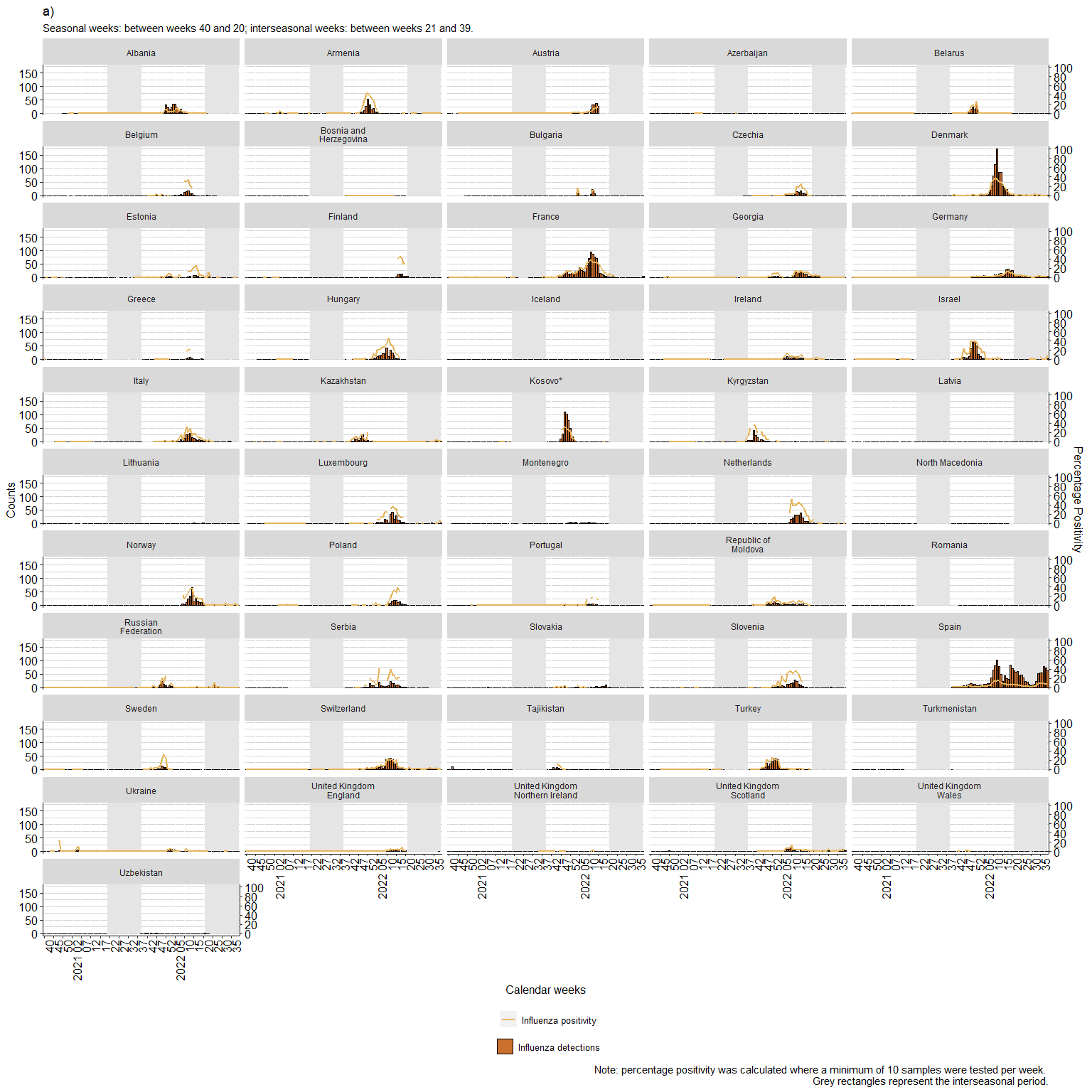


Supplementary Figure 2 (SF2). Individual country-level weekly number and percentage positivity reported for influenza detections through SARI surveillance, between week 40/2020 and week 39/2021, Europe

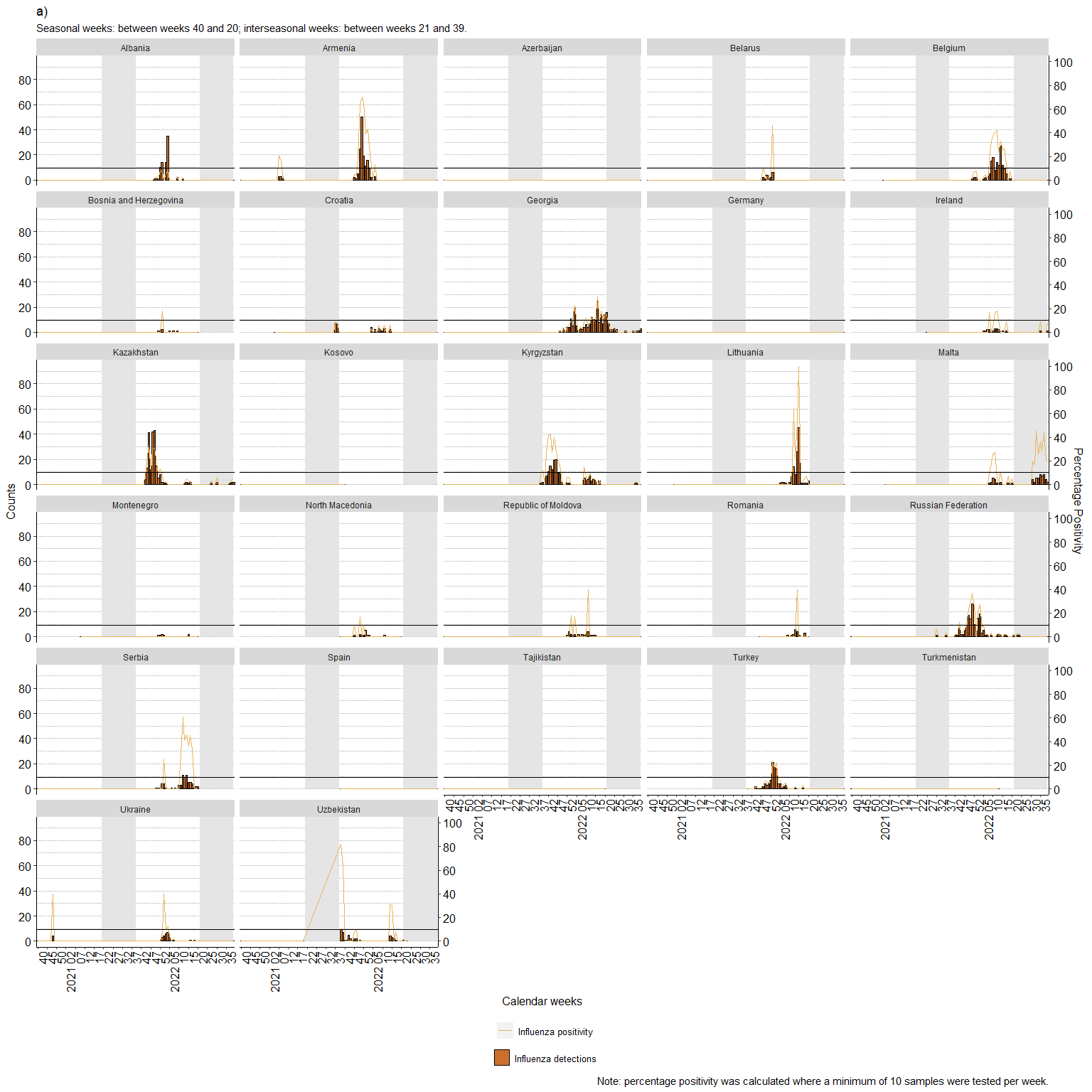


Supplementary Figure 3 (SF3). Individual country-level percentage positivity for influenza, week 40/2020 and 39/2021 in comparison with the mean, minimum, maximum number of specimens in the previous four seasons (week 40 to 39, 2015/16 to 2019/20) through sentinel primary care surveillance, Europe

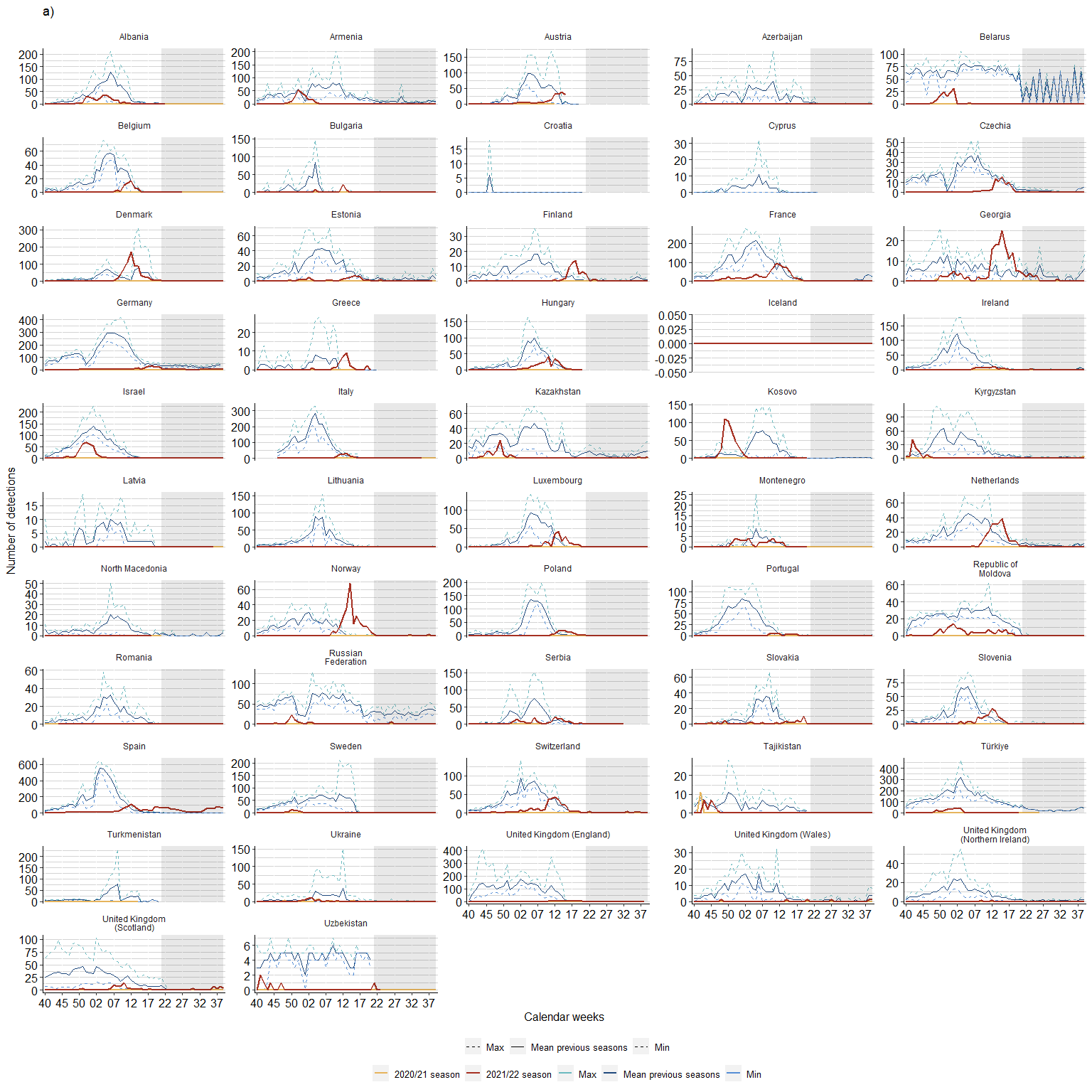


Supplementary Figure 4 (SF4). Individual country-level percentage positivity for influenza, week 40/2020 and 39/2021 in comparison with the mean, minimum, maximum number of specimens in the previous four seasons (week 40 to 39, 2015/16 to 2019/20) through SARI surveillance, Europe

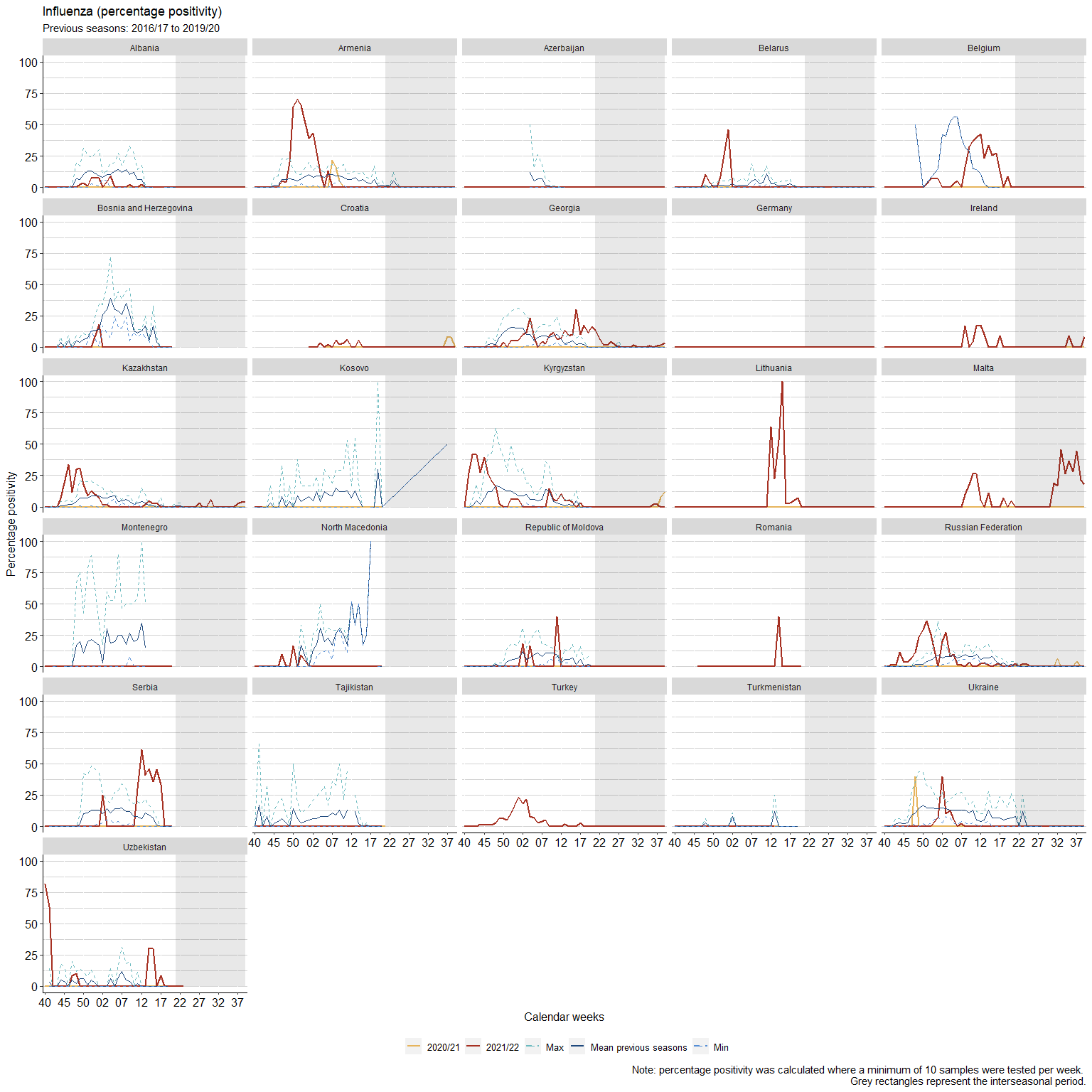

Supplement: Supplementary file 1 — Figure S1. Individual country‐level weekly number and percentage positivity reported for influenza detections through sentinel primary care surveillance, between Week 40/2020 and Week 39/2021, Europe. Figure S2. Individual country‐level weekly number and percentage positivity reported for influenza detections through SARI surveillance, between Week 40/2020 and Week 39/2021, Europe. Figure S3. Individual country‐level percentage positivity for influenza, Week 40/2020 and 39/2021 in comparison with the mean, minimum, maximum number of specimens in the previous four seasons (Week 40 to 39, 2015/16 to 2019/20) through sentinel primary care surveillance, Europe. Figure S4. Individual country‐level percentage positivity for influenza, Week 40/2020 and 39/2021 in comparison with the mean, minimum, maximum number of specimens in the previous four seasons (Week 40 to 39, 2015/16 to 2019/20) through SARI surveillance, Europe. [file IRV-18-e13297-s001.docx]
